# Pancreatic Cancer: From Genetic Mechanisms to Translational Challenges

**DOI:** 10.3390/cancers15164056

**Published:** 2023-08-11

**Authors:** Lorenza Pastorino, Paola Ghiorzo, William Bruno

**Affiliations:** 1 Genetics of Rare Cancers, IRCCS Ospedale Policlinico San Martino, L.go R. Benzi X, 16132 Genoa, Italywilliam.bruno@unige.it (W.B.); 2 Department of Internal Medicine and Medical Specialties, University of Genoa, V.le Benedetto XV, 6, 16132 Genoa, Italy

Pancreatic ductal adenocarcinoma (PDAC), one of the most aggressive malignancies in industrialized countries, is predicted to become the second leading cause of cancer deaths by 2040 [1]. Thereby, there are challenges both in terms of its early detection and treatment. Despite recent advancements in the fields of the molecular genetics and genomics of PDAC, translational challenges remain, and more research is needed. This Special Issue aimed to provide updated research results and future perspectives of the field. To this end, we collected studies and reviews on PDAC tumorigenesis and its translational challenges using molecular genetics approaches. The genetic determinants of inheritance, as well as biomarkers for early diagnosis, the mechanisms of progression and metastasis, and their potential actionability for novel therapeutic strategies, were investigated.

PDAC heritability has historically been associated with hereditary cancer syndromes, and much of the knowledge on the genetics of PDAC has come from the selection of high-risk individuals in clinical practices who underwent molecular diagnostic testing due to them having a suggestive personal or family history of PDAC and related malignancies. Indeed, many germline pathogenic variants (GPVs) in cancer predisposition genes correlating with inherited cancer syndromes, e.g., hereditary breast and ovarian cancer syndrome, have been identified in patients with PDAC. The frequency of *BRCA1/2* GPVs fluctuates between different ethnic groups, and it depends on the selection criteria. Consistent with this, a study by Zampiga et al. confirms the prevalence of *BRCA2* GPVs among PDAC patients with hereditary breast-and-ovarian-cancer-prone families [2]. Dal Buono et al. show that 20% of their cohort of PDAC cases were associated with clinically relevant GPVs in known predisposition genes [3]. The genetic heterogeneity identified in their study reinforces the value of using a multi-gene panel in PDAC genetic testing. Interestingly, they tested 113 consecutively enrolled patients referred to cancer genetic clinics for metastatic PDAC, early-onset PDAC, suspected hereditary syndrome, or a positive family history using a 19-gene cancer panel. They found a 30% (16/55) mutational rate in the subgroup tested for suspected hereditary syndrome (PDAC and other synchronous or metachronous tumors or a suggestive family history).

Although the identification of GPVs in highly suspect individuals with PDAC provides a useful perspective regarding the association between these variants and the risk of developing this cancer, this approach may overestimate this association due to the potential ascertainment bias. A study by Astiazaran-Symonds E et al. [4] addresses this issue by reviewing the prevalence of GPVs in predisposition genes and the risk of developing PDAC, adopting a “genome first” approach.

Overall, the analysis carried out on genomic and phenotypic data from a general population not selected for a specific diagnosis (376,068 individuals, including 1009 with PDAC) found that 2.4% of the general population and 7% of individuals with PDAC were heterozygous for GPVs in *ATM*, *BRCA1*, *BRCA2*, *CDKN2A*, *CHEK2*, and *PALB2*.

These authors also estimated the risk of PDAC due to GPVs in a single gene were the highest for *ATM*, *CDKN2A*, and *PALB2* and the lowest for *CHEK2*.

A previous systematic literature review [5] of data from up to 27 studies including more than 12,000 patients identified *ATM*, *BRCA1*, *BRCA2*, *CDKN2A*, *CHEK2*, and *PALB2* as the genes most frequently (>9% of cases) mutated in unselected pancreatic cancer patients. Overall, this finding supports the strong overlap between genes underlying a susceptibility to different malignancies, while no single gene appears to be predominant for pancreatic cancer.

In the analysis by Puccini et al. [6] of 422 PDAC patients using a 51 gene panel unselected for having a personal or family history, the authors identified GVPs in 17% of cases. Among them, GPVs in *ATM*, *BRCA1*, *BRCA2*, and *CDKN2A* were found in 11.1% of the cases. The most frequent GPVs are in *CDKN2A* (4.5% of cases, all diagnosed above age 50 years), *BRCA2* (3%), and *BRCA1* (1.5%), which were all diagnosed by age 70 years, and *ATM* (2.1%). GPVs in *CHEK2* are found in 1.7% of cases. Forty-one percent of the patients carrying GPVs had no personal or family history suggestive of cancer predisposition syndromes and would have been missed following the classic criteria for access to genetic counseling. The study supports the usefulness of analyzing *CDKN2A* and *ATM* genes in addition to *BRCA1/2*, regardless of patients’ family histories.

The prevalence of GVPs in this study is in line with the results of Astiazaran-Symonds et al. [5], except for *CDKN2A*, which appears to have a higher rate of them; this was probably influenced by a founder effect in the study population [7].

These data also reinforce the utility of using a gene panel sequencing approach to genetic testing in PDAC cases, independent of the patients’ family history, for cascade testing and selection of familial GPV carriers for surveillance protocols. However, PDAC surveillance protocols for GPV carriers are limited to research studies and biomarkers of early detection, as well as progression, are urgently needed.

In the search for early detection biomarkers for PDAC, Kamal MA et al. reported on KRAS-induced secreted proteins, identifying high levels of LAMC2 and PTX3 detected at the early stages (I–IIB) and PDAC patients with low levels of in CA19-9 patients. They suggest that LAMC2 and PTX3 released via systemic circulation may be helpful in selecting patients for future diagnostic imaging [8].

He H. provide an elegant demonstration of the role of the PCSK6 gene as one crucial mediator of tumor progression, suggesting a possible target for tackling liver metastases that frequently occur in PDAC [9].

An interesting new perspective comes from studies on cancer-associated fibroblasts (CAFs), which are key players of the dense, fibrotic stroma characterizing the PDAC microenvironment. Bryce et al. discuss the recent advances in CAFs biology, their evolution and relationship with other cell types, and potential future therapeutic strategies for targeting CAF subtypes. These include leveraging CAF plasticity by switching CAFs to a more tumour-suppressive subtype (converting “bad stroma” to “good stroma”, possibly as an adjunct to immunotherapy or conventional chemotherapy) or targeting systemic inflammation by altering the CAF secretome [10].

However, there are numerous challenges in this process. For example, there is a lack of a universal CAF marker. Nonetheless, as emphasized by the authors, many avenues for future research have been opened, and there is an exciting potential to leveraging novel technologies to profile this previously unknown aspect of PDAC in exceptional detail.

Greco et al. [11], in their review on the epithelial-to-mesenchymal transition (EMT) as a mechanism of pancreatic cancer progression, include EMT among the hallmarks of cancer that could potentially modify our clinical thinking. The translational improvement provided by the identification of EMT markers suitable for deciphering the aggressive behavior of PDAC could eventually modify the clinical scenario, possibly contributing to in the advanced diagnosis and monitoring of its evolution and responsiveness to treatments. Despite the evidence supporting the activation of the EMT during cancer progression, our understanding of the relationship between the tumor microenvironment and EMT is not yet mature enough for clinical applications. However, EMT markers in blood from pancreatic cancer migratory cell, epithelial, mesenchymal, and/or hybrid populations expressing both epithelial and mesenchymal-specific genes could represent a significant potential for anticipating PDAC detection, possibly providing a new tool for personalized medicine [11].

Similarly, since an altered metabolism is one of the main driving forces of pancreatic cancers’ development, progression, and response to treatments, new interventions may come from studying lncRNAs, which are frequently aberrantly expressed in PDAC and implicated in metabolic homeostasis. Dalmasso et al. [12] assess the burden of lncRNA dysregulation in pancreatic cancer metabolic reprogramming and point out its effect on this tumor’s natural course and response to treatment.

The challenge of the PDAC response to treatment is reviewed by He Q et al. [12]. PDAC is one of the most intractable malignant tumors worldwide and is known for its refractory nature and poor prognosis, as demonstrated by the fatality rate, which can reach over 90%. Since *KRAS* is one of the most frequently occurring oncogenic mutations in PDAC, the introduction of its inhibitors has opened a new avenue for the clinical treatment of PDAC, considering that KRAS proteins have maintained the reputation of being “undruggable” due to their special molecular structures and biological characteristics, making therapy targeting downstream genes challenging. However, although inhibitors against KRAS mutations have been developed, this therapeutic approach is not routinely used for PDAC patients.

He Q et al. [13] discuss new approaches regarding the *KRASG12C* inhibitor, Sotorasib, which is FDA-approved for patients suffering from *KRASG12C*-driven cancers, and the increasing attention given to the development of inhibitors for other *KRAS* mutations (*G12D/V*) and their potential applications. However, *KRASG12C* mutations account for a tiny proportion of *KRAS* mutations in PDAC, and *KRASG12C* inhibitors currently used in clinical practice have minimal efficacy in PDAC patients. Fortunately, *KRASG12D* inhibitors have been developed and put into preclinical trials, while *KRASG12V* inhibitors are also being explored. It is believed that in the near future, *KRASG12D/V* inhibitors will provide a new perspective of PDAC treatments.

Ma Y et al. [14] investigate the anti-tumor efficacy of two KRAS inhibitors, Sotorasib (KRAS G12C inhibitor) and BI-3406 (KRAS:SOS1 inhibitor), alone or in combination with MEK1/2 inhibitor trametinib and/or PI3K inhibitor buparlisib in seven PDAC cell lines. They demonstrate that the inhibition of KRAS, MEK, and PI3K shows synergistic anti-tumor effects. They also confirm that the inhibition of KRAS and its downstream pathways is a potential novel therapeutic approach for PDAC, providing fundamental data for future in vivo evaluations.

Many of these approaches have not been clinically applied yet, but they represent promising avenues of investigation for deciphering the molecular genetics of PDAC and its potential translational challenges.
